# Sodium Intake and Sodium to Potassium Ratio among New York City Adults in the 2018 Heart Follow-Up Study

**DOI:** 10.1016/j.cdnut.2024.102143

**Published:** 2024-03-21

**Authors:** Christine Dominianni, Divya Prasad, Andrea Sharkey, Elizabeth Solomon, Amber Levanon Seligson, John P Jasek

**Affiliations:** 1 Bureau of Chronic Disease Prevention, New York City Department of Health and Mental Hygiene, Long Island City, NY, United States; 2 Bureau of Epidemiology Services, New York City Department of Health and Mental Hygiene, Long Island City, NY, United States

**Keywords:** sodium, potassium, diet quality, sodium reduction initiatives, access to healthy food

## Abstract

**Background:**

High sodium and low potassium intake are positively associated with blood pressure, a significant risk factor for cardiovascular disease. The mean intake of sodium among United States adults exceeds the chronic disease risk reduction level of 2300 mg/d, whereas potassium intake remains lower than the recommended levels. From 2008 through 2019, there were several local and national initiatives to reduce sodium in New York City (NYC).

**Objectives:**

We aimed to update and compare estimates of sodium intake among NYC adults overall and by covariates from the 2010 Heart Follow-Up Study (HFUS) with the 2018 HFUS. We also estimated the 2018 sodium-to-potassium ratio to understand overall diet quality among demographic groups.

**Methods:**

This cross-sectional study used sodium and potassium measurements from 24-h urine collection and self-reported data from 2509 and 1656 participants in the 2018 and 2010 HFUS, respectively. The weighted mean daily intake of sodium and the sodium-to-potassium ratio were estimated. *T-*tests and multivariable linear regression models with tests for interactions were used to compare changes in sodium intake.

**Results:**

The mean sodium intake of adult New Yorkers in 2018 was 3292 mg/d. Sodium intake did not change from 2010 (3234 mg/d, *P* = 0.45) to 2018 in the overall population, although there was a decrease in sodium intake among adults 18–24 y old (3445 mg/d to 2957 mg/d, *P* = 0.05). The daily mean sodium-to-potassium ratio was 1.7 mg/mg. The highest sodium-to-potassium ratios were among Black females 18–44 y old (2.0) and 45–64 y old (2.2) and Black (2.1) and Latino (2.1) males between 18 and 44 y old.

**Conclusions:**

The lack of population-level changes in sodium intake and the high sodium-to-potassium ratios among Black females and younger Black and Latino males suggest that further efforts to reduce sodium in the food supply and address persistent inequities are needed.

## Introduction

Healthy eating patterns are beneficial to lowering risk of cardiovascular disease (CVD) [1], the leading cause of death in New York City (NYC) [2] and the United States [3]. Increased blood pressure, a significant risk factor for CVD [4], has been positively associated with the consumption of sodium [5] or salt and negatively associated with the consumption of potassium [6]. The USDA, via the Dietary Guidelines for Americans 2020–2025, recommends that individuals 14 y and older limit sodium intake to <2300 mg/d [7]. The National Academies sets the chronic disease risk reduction (CDRR) level, the level above which intake reduction is expected to reduce chronic disease risk within an apparently healthy population, to 2300 mg/d for sodium [8]. As of 2014, the average intake of sodium based on 24-h urine collection was 3608 mg/d among United States adults [9], far exceeding the recommended limit and CDRR. The average potassium intake of 2155 mg/d estimated from 24-h urine collection [9] also did not meet the recommended intake (2300–3400 mg/d depending on sex and life-stage) [7].

In 2010, the Heart Follow-Up Study (HFUS) was conducted using the gold standard measure of 24-h urine collection to assess sodium [10], as well as potassium. The primary goal was to understand the dietary intake of sodium and potassium among NYC adults given nationally representative data at the time were based on 24-h dietary recall, which misses sodium intake from salt added at the table and during food preparation [11]. Data from the 2010 HFUS showed that the average daily intake of sodium exceeded 3200 mg [12], which is well over the current CDRR. Prior to and following the 2010 HFUS, there were several local and national initiatives to help reduce sodium in the food supply and support lower sodium consumption. In 2009, the National Salt Reduction Initiative (NSRI) was launched to reduce sodium in packaged and restaurant foods. From 2010 to 2021, the Centers for Disease Control and Prevention funded several sites across the United States, including NYC, through the Sodium Reduction in Communities Program (SRCP) to implement sodium reduction strategies. In 2011, the USDA and Food and Drug Administration (FDA) invited the public to comment on strategies to reduce sodium consumption [13]; and in 2016, the FDA released draft 2- and 10-y voluntary sodium reduction guidance [14]. Locally, in 2008, the NYC Food Standards were established to apply evidence-based nutrition criteria, including sodium limits, to foods served by NYC government agencies and their subcontractors. SRCP funding supported voluntary adoption of the NYC Food Standards, including in private hospitals in NYC through the Healthy Hospital Food Initiative, which was launched in 2012. Citywide media campaigns ran in 2010 and 2013 to encourage people to choose lower sodium items. In 2016, the sodium warning rule went into effect in chain restaurants requiring an icon on menus for items containing ≥2300 mg sodium [15]. A hypertension outreach and education campaign targeting primary care providers and staff across NYC also ran from November 2017 through April 2019 promoting hypertension diagnosis and management, including incorporating low-sodium diet counseling, among their patients. A second wave of HFUS was conducted in 2018 to provide updated estimates of sodium intake in NYC, which could facilitate the evaluation of the initiatives collectively.

Findings from the 2010 HFUS additionally showed that sodium consumption was higher among Black and Latino/a New Yorkers compared with White New Yorkers [12]. Potassium intake was also found to be low among NYC adults overall, especially among Black, Latino/a, and Asian/Pacific Islander New Yorkers and those living in households with low-income levels [16]. Structural racism has historically driven and continues to drive unequal distribution of resources that are the root causes of unjust health inequities [17]. For example, fast-food restaurants, where high-sodium foods can be more easily purchased, tend to be concentrated in Black and Latino/a communities and neighborhoods with a higher prevalence of households with lower incomes across the United States, whereas potassium-rich foods, such as fresh fruits and vegetables, are less accessible because of high cost or fewer grocery stores and supermarkets [[18], [19], [20]]. The same communities often bear the burden of hypertension and health conditions that hypertension can cause [[21], [22], [23]], suggesting that ongoing inequities in access to healthy food contribute to and reinforce existing health disparities.

The primary objective of this study was to update estimates of sodium intake among NYC adults overall and by demographic, socioeconomic, and lifestyle factors and assess if there were any changes in NYC adults’ consumption of sodium since 2010, especially because there were multiple efforts to reduce sodium consumption during this period. A secondary objective of the study included an assessment of the sodium-to-potassium ratio to understand how diet quality varies across different populations, particularly by race and ethnicity. A greater ratio indicates poorer diet quality, because of higher sodium intake and/or lower potassium intake [24]. This article shares the findings from HFUS to inform programs and policies aimed at reducing sodium intake and increasing potassium intake with the goal of reducing New Yorkers’ blood pressure and minimizing their risk for CVD through equitable access to healthy foods for all.

## Methods

### Sample

HFUS was a population-based surveillance study of sodium and potassium intake among NYC adults conducted by the NYC Department of Health and Mental Hygiene (DOHMH) in 2010 and 2018. The Institutional Review Board of the NYC DOHMH approved the study.

To obtain a representative sample of adult New Yorkers (18 y and older), we recruited a subset of participants from the NYC Community Health Survey (CHS). CHS is an annual survey of 8000–10,000 NYC adults conducted by the NYC DOHMH that captures data on multiple aspects of health conditions and dietary and lifestyle behaviors. From 2002 through 2020, CHS was a telephone survey; a cell phone sample frame was added in 2009 to the existing landline sample frame. CHS includes respondents from noninstitutionalized, residential households across NYC [25].

The 2018 HFUS sample was intended to come entirely from the 2018 CHS sample but delays limited the number of participants who could be recruited into HFUS during that calendar year. CHS participants were recruited for HFUS from July 31, 2018, to December 19, 2018. A supplemental random digit dial (RDD) sample of participants selected from active cell phone records was recruited from September 25, 2018, to April 3, 2019, to increase sample size and statistical power. Individuals were considered ineligible for participation if they were pregnant, currently breastfeeding or lactating (asked of females only), or receiving kidney dialysis in the 12 mo prior to completing the eligibility screener. Out of 5068 CHS participants who were screened for eligibility, 1772 were deemed eligible and went on to complete a phone-based follow-up interview with questions about CVD, hypertension, and nutrition. Among the RDD sample, 4980 people were screened for eligibility of which 2412 were deemed eligible and went on to complete a longer version of the follow-up interview that also included select questions from the 2018 CHS.

Kits with instructions for collecting 24-h urine samples were sent to respondents for whom home visits could be scheduled. Medical technicians picked up the urine samples, and measured blood pressure [26], weight, and height from 1230 CHS and 1526 RDD participants who completed the appointment. At the home visit, the 24-h urine samples were determined to be invalid if the volume was <500 ml or the participant reported including the first void of the day in the collection; these participants were asked to redo the 24-h urine collection (*n =* 62). The urine samples from 1219 CHS and 1522 RDD participants were sent to the laboratory for the analysis of sodium and potassium, as well as albumin and creatinine, and subjected to additional completeness criteria. A total of 229 participants were excluded if any of their 4 analyte levels were outside the expected ranges, collection time was missing, normalized urine volume was <500 mL, creatinine levels were <6.05 mmol for males or <3.78 mmol for females, the participant self-reported missing a collection, or the collection time was <18 h (based on the minimum duration of collection that occurred in the 2010 HFUS). The final analytic sample was 2512 (CHS-derived *n =* 1110 and RDD-derived *n =* 1402, Supplemental Figure 1).

The 2010 HFUS sample, briefly, was recruited from 5830 eligible participants who completed the 2010 CHS. From the 2305 participants who went on to complete the phone-based follow-up interview, 24-h urine samples were provided and met validity criteria for a final analytic sample of 1656. Additional details for the 2010 study can be found elsewhere [27].

### Sodium, potassium, and the sodium-to-potassium ratio

Urinary sodium and potassium were measured using the ion-selective electrode and atomic absorption spectroscopy methods on Beckman Coulter AU Analyzers. Laboratory values were normalized to 24 h based on each participant’s total collection time. For example, if the duration of collection was 23 h, we multiplied the sodium value by 24/23. Results are presented for normalized values of sodium and potassium (mg/d) and the sodium-to-potassium ratio that was calculated by dividing the normalized sodium value (mg) by the normalized potassium value (mg).

### Covariates

Covariates were selected per prior analysis of the 2010 HFUS data [12] except for diet quality (not asked in 2018). BMI was calculated by dividing participant weight (kg) by squared height (m) measured at the home visits. Participants were then assigned to 1 of 4 groups: adults with underweight (<18.5 kg/m^2^), normal weight (18.5–24.9 kg/m^2^), overweight (25–29.9 kg/m^2^), or obesity (≥30 kg/m^2^). All other information came from self-reported responses to questions in the CHS or the follow-up interview and were grouped as follows: age (18–24, 25–44, 45–64, ≥65), country of birth [United States (including Puerto Rico and United States territories), Outside of United States], physical activity in the past 30 d (yes, no), and sex assigned at birth (male, female). In 2010, respondents were asked to confirm whether they were male or female and not the sex assigned on their birth certificate. Recoded variables included smoking status (never, current, former), heavy drinking defined as males having >2 drinks/d or females having >1 drink/d (yes, no), household income (<200%, 200%–399%, or ≥400% of the federal poverty level), history of CVD (yes, no), and race and ethnicity. Participants were assigned a race and ethnicity category based on their responses to a single Hispanic ethnicity question (yes, no) followed by a multicategory race question (White, Black or African American, Asian, Middle Eastern or North African, Native Hawaiian or Other Pacific Islander, American Indian, or Alaska Native) with options to select multiple categories and to verbally specify another race not represented in the preset categories. Final categories for race and ethnicity included Latino/a or Hispanic (regardless of their selected race), Asian/Pacific Islander (including Native Hawaiian), Black, White (including Middle Eastern or North African), and Other (including those who selected multiple race categories). The 2010 question on race did not include “Middle Eastern or North African” as an option. The history of CVD was defined using different variables between years. The 2010 survey included 6 questions about hypertension, congestive heart failure, coronary artery disease, angina pectoris, myocardial infarction, and stroke. The 2018 survey included 3 questions about hypertension, stroke, and heart disease. When we combined congestive heart failure, coronary artery disease, angina pectoris, and myocardial infarction into an indicator of heart disease in the 2010 sample, we found estimates of heart disease were comparable between 2010 (8.3%) and 2018 (8.4%). As such, we assumed that the 2018 question on heart disease captured congestive heart failure, coronary artery disease, angina pectoris, and myocardial infarction. Additionally, the hypertension question changed so that female respondents were asked to not include hypertension related to pregnancy in the 2018 survey. However, given the relationship between hypertension and sodium, we thought it was best to keep this variable in the analysis. Our analytic definition of having a history of CVD included anyone who reported hypertension, stroke, or heart disease (congestive heart failure, coronary artery disease, angina pectoris, or myocardial infarction for 2010). The 2010 survey also included a question on transient ischemic attack that was not asked in the 2018 survey so was not included in the CVD history definition.

We additionally included variables to assess the awareness of sodium content in packaged foods and the frequency of eating meals prepared outside the home in both years. Sodium content awareness was based on the question “When you look at the Nutrition Facts panel to decide about a food product, how often do you look for information about sodium or salt content?” and categorized as “most of the time to always,” “rarely to sometimes,” “never,” or “does not use the Nutrition Facts panel at all.” The frequency of eating meals prepared outside the home was based on the question “How often do you eat meals that were prepared at a restaurant, deli, or street vendor? Please include meals eaten at a restaurant, carried out or delivered” and categorized as 0, >0−3, >3–6, or >6 meals/wk.

### Statistical analysis

The 2018 dataset included 2 independent samples, CHS and RDD, that were weighted separately to the NYC adult, noninstitutionalized population and then combined and adjusted to create a final weighted dataset. Unweighted and weighted age-adjusted frequencies and percentages of all covariates are reported for the 2010 and 2018 samples. Unweighted estimates reflect the characteristics of the study samples, whereas the weighted estimates reflect the characteristics of the NYC adult population in each year. Weighted estimates were compared between 2010 and 2018 using *T*-tests.

We assessed the normality of the sodium and potassium value distributions prior to analysis. We defined outliers for the 2018 dataset if they were greater than the maximum values for sodium (≥15,000 mg/d) and potassium (≥12,000 mg/d) in the 2010 dataset. After excluding the outliers in 2018, the final sample sizes were 2509 for the analysis of sodium and 2511 for the analysis of potassium. We present sodium data as a weighted mean daily intake of sodium and a weighted percent of NYC adults who consumed less than the sodium CDRR (<2300 mg/d). Each estimate type was calculated for the overall sample and for each category of study covariates for both years. We used *T*-tests to compare means and percentages between categories of covariates in 2010 and 2018 and within categories in each year. Overall estimates of potassium and the sodium-to-potassium ratio and stratified estimates of this ratio by race and ethnicity, age, and sex were also calculated. All estimates were age-adjusted to the United States 2000 Standard Population unless they were age-stratified estimates.

To evaluate for changes in sodium consumption between 2010 and 2018 after further adjustment, we constructed multivariable linear regression models combining the 2010 and 2018 datasets. Overall differences were assessed using *T*-tests, whereas changes across categories of each covariate were assessed using tests for interaction between year and each covariate. All models included age, sex, race and ethnicity, country of birth, household income, BMI, smoking status, physical activity, heavy drinking, Nutrition Facts panel, eating meals purchased at a deli, street vendor, or restaurant, CVD history, and mean daily potassium intake. Weighted mean sodium and *P* values are reported for each category of all categorical covariates. Missing values for all covariates were not included in multivariable regression models. To assess whether the skewed values of sodium and potassium affected associations, we also ran the multivariable regression models using log-transformed values of sodium and potassium. We analyzed data with SUDAAN software version 11.0.3 (Research Triangle Institute).

## Results

### Characteristics of HFUS study sample and NYC population changes

In 2018, most survey respondents (unweighted: 75%) were between the ages of 25 and 64 and slightly over half (54%) of respondents were female (Table 1). Over one third (36%) of respondents were Latino/a, 29% were White, 24% were Black, 8% were Asian/Pacific Islander, and the remaining 3% were of Other race or ethnicity. The 2010 and 2018 samples were weighted to be representative of the NYC adult population in their respective year; between years, there were shifts toward more adults who never smoked (66% in 2018 compared with 59%, in 2010 *P* = 0.002) and fewer adults who formerly smoked (19% compared with 23%, *P* = 0.03). There were also decreases in using the Nutrition Facts panel for information about sodium content most of the time or always (33% compared with 41%, *P* < 0.001) and increases in eating >6 meals prepared outside the home per week (18% compared with 7%, *P* < 0.001).

**TABLE 1:** Demographic, socioeconomic, lifestyle, and health factors of NYC adults (≥18 y old) in the NYC Heart Follow-Up Study, 2010 and 2018

	2010		2018		2018 vs. 2010
	Unweighted % (*N*)	Weighted %	Unweighted % (*N*)	Weighted %	*P* value^1^
Total	1656		2512		
Age (y)					
18–24	6 (102)	13	6 (142)	13	0.94
25–44	30 (504)	43	35 (879)	40	0.20
45–64	42 (701)	28	40 (994)	31	0.08
≥65	21 (348)	15	20 (497)	15	0.90
Sex					
Male	42 (692)	46	46 (1153)	46	0.90
Female	58 (964)	54	54 (1359)	54	0.90
Race/ethnicity					
Asian/Pacific Islander	5 (84)	10	8 (206)	13	0.08
Black	27 (440)	23	24 (594)	22	0.51
Latino/a	29 (485)	24	36 (900)	27	0.21
Other	2 (35)	4	3 (84)	3	0.23
White	37 (612)	39	29 (728)	36	0.16
Country of birth					
United States	63 (1046)	57	57 (1434)	58	0.70
Outside of United States	37 (609)	43	43 (1072)	42	0.70
Household income (% of FPL)					
<200	48 (720)	53	53 (1332)	50	0.18
200–399	18 (267)	16	16 (412)	17	0.84
≥400	35 (520)	30	31 (768)	33	0.20
BMI (kg/m^2^)					
<18.5 (underweight)	2 (39)	3	1 (30)	2	0.19
18.5–24.9 (normal weight)	32 (521)	32	29 (733)	33	0.48
25–29.9 (overweight)	34 (566)	35	34 (845)	34	0.70
≥30 (obesity)	32 (526)	31	35 (884)	31	0.88
Smoking status					
Never	56 (918)	59	62 (1537)	66	0.002
Current	17 (287)	18	16 (389)	14	0.08
Former	27 (443)	23	23 (572)	19	0.03
Physically active in past 30 d					
Yes	76 (1257)	74	73 (1839)	74	0.86
No	24 (399)	26	27 (671)	26	0.86
Heavy drinking^2^					
Yes	5 (82)	6	5 (128)	5	0.76
No	95 (1559)	94	95 (2362)	95	0.76
Look for information about sodium when using the Nutrition Facts panel					
Most of the time to always	46 (763)	41	38 (957)	33	<0.001
Rarely to sometimes	28 (467)	29	31 (761)	34	0.02
Never	9 (153)	11	8 (198)	9	0.09
Never use the Nutrition Facts panel	16 (268)	19	23 (580)	24	0.02
Eat meals purchased at deli, street vendor, or restaurant					
0 meals/wk	25 (411)	22	23 (568)	20	0.31
>0–3 meals/wk	59 (976)	60	51 (1279)	50	<0.001
>3–6 meals/wk	10 (171)	10	10 (255)	11	0.54
>6 meals/wk	6 (92)	7	16 (400)	18	<0.001
History of cardiovascular disease^3^					
Yes	37 (598)	32	36 (890)	29	0.13
No	63 (1042)	68	64 (1613)	71	0.13

Abbreviation: FPL, federal poverty level.

1 *P* values were obtained from *t*-test comparisons of the 2010 and 2018 weighted percentages.

2 Defined as men having >2 drinks/d or females having >1 drink/d.

3 Includes self-reported hypertension, self-reported stroke, or self-reported heart disease. Note that the 2010 definition was based on separate responses about self-reported congestive heart failure, coronary artery disease, angina pectoris, or myocardial infarction, which we considered to be captured by the broader 2018 question on heart disease.

### Sodium intake in 2018 and compared with sodium intake in 2010

In 2018, the mean and median intake of sodium among adult New Yorkers was 3,292 mg/d and 3009 mg/d (IQR: 2115–4096 mg/d), respectively (Table 2). The mean intake did not change from the mean intake in 2010 (mean = 3234 mg/d, *P =* 0.45). Among all adults, 31% consumed less than the sodium CDRR (2300 mg/d), which also did not change from 2010 (31%, *P =* 0.75, Table 3). There were decreases in the mean sodium intake among adults who were 18–24 y old (2957 mg/d compared with 3445 mg/d in 2010, *P =* 0.05) or who drank heavily (3038 mg/d compared with 3674 mg/d, *P =* 0.05). Increases in sodium were observed among adults who were 25–44 y old (3537 mg/d compared with 3248 mg/d in 2010, *P =* 0.04) or had a history of CVD (3601 mg/d compared with 3096 mg/d, *P =* 0.02). Tests for the interaction between year and each covariate from fully adjusted models indicated significant changes in sodium intake by age (*P =* 0.02) and by the frequency of eating meals prepared outside the home (*P =* 0.03) (Table 2). The interaction between year and frequency of eating meals prepared outside the home was no longer significant (*P =* 0.07) when we repeated regression analyses using log-transformed sodium values (Supplemental Table 1). The percentage of adults consuming less than the sodium CDRR also did not change from 2010 to 2018 by age, sex, or race and ethnicity (Table 3).

**TABLE 2:** Age-adjusted and fully adjusted weighted mean sodium intake of NYC adults (≥18 y old), 2010 and 2018

	Age-adjusted^1^			Fully adjusted^2^		
	2010 Weighted mean sodium (mg/d) (SE)	2018 Weighted mean sodium (mg/d) (SE)	*P* value^3^	2010 Weighted mean sodium (mg/d) (SE)	2018 Weighted mean sodium (mg/d) (SE)	*P* value^4^
Total	3234 (59)	3292 (48)	0.45	3273 (60)	3295 (43)	0.76
Age (y)						
18–24	3445 (193)	2957 (152)^8^	0.05	3857 (191)	3265 (140)	0.02
25–44 (Ref)	3248 (107)	3537 (86)	0.04	3288 (113)	3494 (83)	
45–64	3467 (92)	3375 (77)	0.44	3303 (92)	3252 (69)	
≥65	2630 (91)^8^	2821 (83)^8^	0.12	2748 (104)	2873 (84)	
Sex						
Male (Ref)	3562 (92)	3788 (78)	0.06	3501 (97)	3671 (74)	0.07
Female	2949 (72)^8^	2862 (50)^8^	0.32	3075 (74)	2969 (50)	
Race/ethnicity						
Asian/Pacific Islander	3059 (211)	3198 (130)	0.58	3443 (275)	3374 (147)	0.43
Black	3409 (130)	3268 (105)	0.40	3582 (119)	3382 (95)	
Latino/a	3387 (111)	3453 (78)	0.63	3457 (101)	3479 (66)	
Other	3087 (245)^7^	3099 (189)	0.97	3150 (218)	3248 (150)	
White (Ref)	3146 (97)	3233 (105)	0.55	2923 (102)	3099 (86)	
Country of birth						
United States (Ref)	3278 (77)	3250 (64)	0.78	3334 (87)	3311 (59)	0.45
Outside of United States	3146 (90)	3329 (72)	0.11	3187 (85)	3273 (68)	
Household income (% of FPL)						
<200	3305 (92)	3340 (74)	0.76	3351 (85)	3396 (69)	0.50
200–399	3342 (150)	3315 (118)	0.89	3350 (125)	3198 (115)	
≥400 (Ref)	3304 (128)	3193 (70)	0.45	3111 (114)	3189 (71)	
BMI (kg/m^2^)						
<18.5 (underweight)	2877 (414)^7^	2395 (148)^7^^,^^8^	0.27	3635 (596)	2783 (209)	0.08
18.5–24.9 (normal weight) (Ref)	3011 (99)	2857 (62)	0.19	3082 (99)	2898 (68)	
25–29.9 (overweight)	3117 (95)	3310 (84)^8^	0.13	3140 (104)	3278 (74)	
≥30 (obesity)	3602 (120)^8^	3815 (102)^8^	0.18	3598 (108)	3756 (87)	
Smoking status						
Never (Ref)	3137 (73)	3267 (61)	0.17	3220 (85)	3262 (56)	0.79
Current	3429 (139)	3317 (104)	0.52	3401 (138)	3319 (94)	
Former	3344 (147)	3414 (141)	0.73	3328 (113)	3377 (100)	
Physically active in past 30 d						
Yes (Ref)	3150 (66)	3287 (53)	0.11	3219 (70)	3303 (49)	0.17
No	3479 (123)^8^	3296 (101)	0.25	3425 (117)	3277 (89)	
Heavy drinking^5^						
Yes	3674 (271)	3038 (175)	0.05	3608 (255)	3171 (141)	0.10
No (Ref)	3203 (60)	3304 (50)	0.19	3252 (62)	3302 (45)	
Look for information about sodium when using the Nutrition Facts panel						
Most of the time to always (Ref)	3228 (97)	3234 (85)	0.96	3212 (98)	3187 (73)	0.66
Rarely to sometimes	3126 (106)	3208 (79)	0.53	3284 (106)	3271 (74)	
Never	3164 (116)	3476 (135)	0.08	3197 (125)	3420 (125)	
Never use the Nutrition Facts Panel	3486 (155)	3420 (104)	0.72	3389 (152)	3467 (97)	
Eat meals purchased at deli, street vendor, or restaurant						
0 meals/wk (Ref)	3095 (141)	3286 (127)	0.32	3042 (139)	3222 (106)	0.03
>0–3 meals/wk	3290 (80)	3222 (69)	0.52	3405 (80)	3249 (59)	
>3–6 meals/wk	3091 (118)	3388 (122)	0.08	3258 (102)	3375 (122)	
>6 meals/wk	3472 (208)	3345 (100)	0.58	2920 (235)	3447 (104)	
History of cardiovascular disease^6^						
Yes	3096 (133)	3601 (172)^8^	0.02	3260 (114)	3367 (86)	0.44
No (Ref)	3202 (67)	3244 (56)	0.63	3280 (73)	3266 (54)	

Abbreviation: FPL, federal poverty level.

1 Age-adjusted weighted means and SEs are shown.

2 Fully adjusted weighted means and SEs from models that included year, year×covariate interaction, age, sex, race/ethnicity, country of birth, household income, BMI, smoking status, physically active, heavy drinking, look for sodium when using the Nutrition Facts panel, eat meals purchased at deli, street vendor, or restaurant, cardiovascular disease history, and daily potassium intake.

3 *P* values were obtained from *t*-test comparisons of the 2010 and 2018 mean values.

4 *P* values were obtained from *t*-test comparisons of 2010 and 2018 overall and from tests for interaction between year and all other variables.

5 Defined as men having >2 drinks/d or females having >1 drink/d.

6 Includes self-reported hypertension, self-reported stroke, or self-reported heart disease. Note that the 2010 definition was based on separate responses about self-reported congestive heart failure, coronary artery disease, angina pectoris, or myocardial infarction, which we considered to be captured by the broader 2018 question on heart disease.

7 Estimate should be interpreted with caution. Estimate’s relative SE is >30% or the sample size is too small making the estimate potentially unreliable.

8 *P <* 0.05 when compared with the reference group.

**TABLE 3:** Percentage of NYC adults (≥18 y old) consuming less than the CDRR level for sodium, 2010 and 2018

	2010 Weighted % (95% CI)^1^	2018 Weighted % (95% CI)^1^	2018 vs. 2010 *P* value^2^
Total	31 (28, 35)	31 (28, 33)	0.75
Age (y)			
18–24	27 (17, 42)^3^	34 (25, 45)	0.39
25–44	30 (25, 37)	27 (23, 31)	0.32
45–64	25 (20, 30)	28 (24, 32)	0.33
≥65	48 (40, 56)	42 (35, 49)	0.23
Sex			
Male	24 (20, 29)	20 (17, 24)	0.18
Female	38 (33, 43)	39 (36, 43)	0.56
Race/ethnicity			
Asian/Pacific Islander	34 (22, 49)^3^	31 (24, 39)	0.73
Black	30 (24, 37)	32 (26, 38)	0.73
Latino/a	28 (22, 35)	27 (23, 32)	0.83
Other	22 (10, 42)^3^	25 (16, 38)^3^	0.75
White	32 (27, 38)	33 (28, 39)	0.75

Abbreviations: CDRR, chronic disease risk reduction; CI, confidence interval.

1 Age-adjusted weighted percentages and CIs are shown.

2 *P* values were obtained from *t*-test comparisons of the 2010 and 2018 age-adjusted weighted percentages.

3 Estimate should be interpreted with caution. Estimate’s relative SE is >30%, or the 95% CI half-width is >10, or the sample size is too small, making the estimate potentially unreliable.

In 2018, we found that sodium intake was higher among adults who were between 25 and 44 y old (3537 mg/d) compared with adults 18–24 y old (2957 mg/d, *P <* 0.01) or ≥65 y old (2821 mg/d, *P <* 0.01) and among males (3788 mg/d) compared with females (2862 mg/d, *P <* 0.01) (Table 2). Adults with overweight (3310 mg/d, *P <* 0.01) and adults with obesity (3815 mg/d, *P <* 0.01) also had higher intakes of sodium compared with adults with normal weight (2857 mg/d).

### Sodium intake stratified by age, sex, and race and ethnicity and in relation to potassium intake in 2018

**FIGURE 1:**
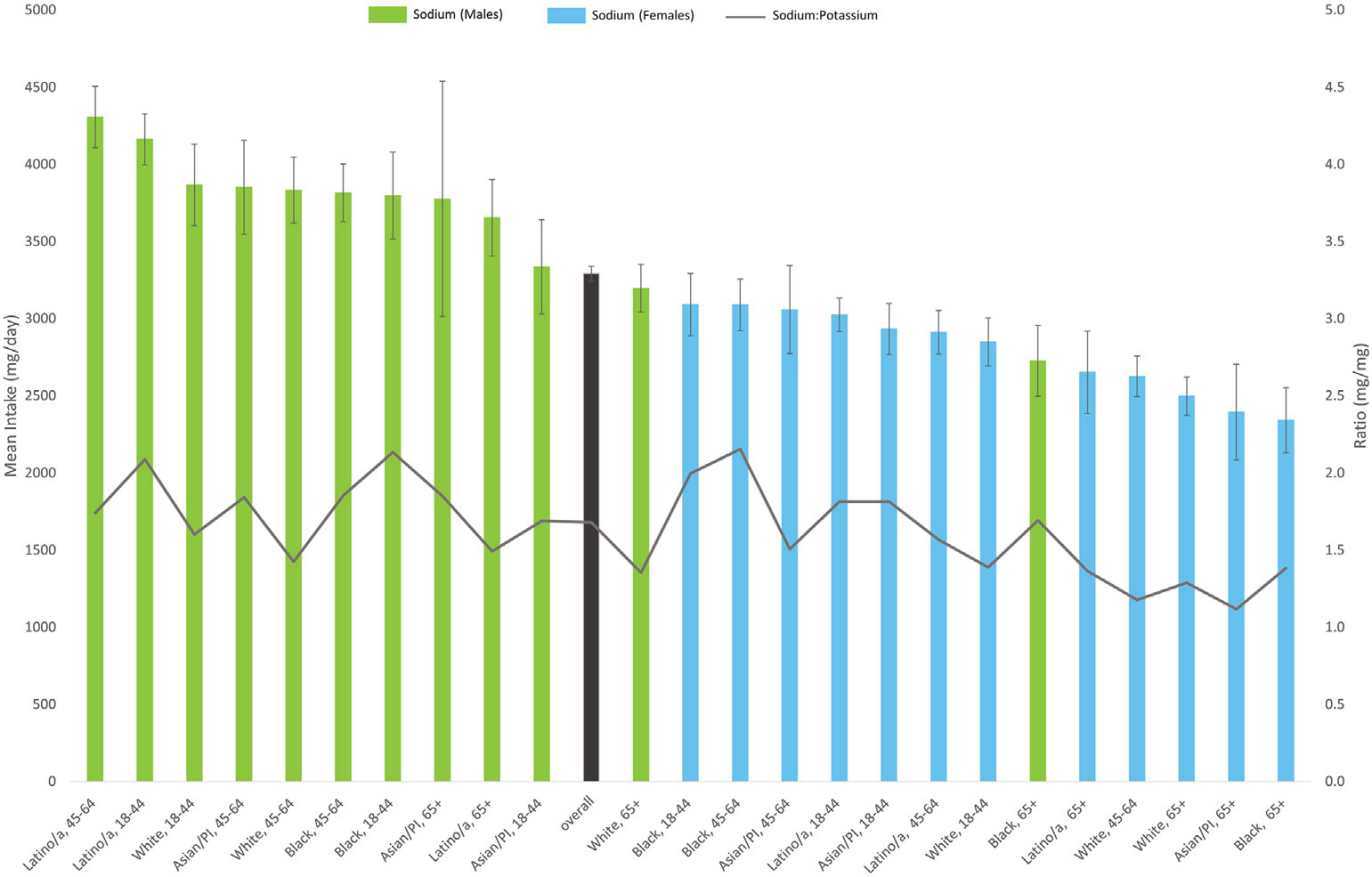
Weighted mean (SE) sodium intake and weighted mean sodium to potassium ratio among NYC adults (≥18 y old) by race and ethnicity, age, and sex in the NYC Heart Follow-Up Study, 2018.

Three-way stratified analysis by age, sex, and race and ethnicity further showed that Latino males between the ages of 45 and 64 y old (4307 mg/d) and 18 and 44 (4163 mg/d) y old had the highest mean intake of sodium, but these groups were generally not significantly different when compared with males of the same age across other racial and ethnic groups (Figure 1, Supplemental Table 2). The daily mean intake of potassium was 2198 mg/d and the daily mean sodium-to potassium ratio was 1.7 mg/mg. The highest sodium-to-potassium ratios were among Black females between 18 and 44 y old (2.0) and 45 and 64 y old (2.2) and Black (2.1) and Latino (2.1) males between 18 and 44 y old.

## Discussion

We found that in NYC, the average intake of sodium was 3292 mg/d in 2018 and that the majority (69%) of New Yorkers continue to have a sodium intake well above the sodium CDRR of 2300 mg/d. Sodium intake did not change from 2010 to 2018 in the overall population, although it decreased among adults between 18 and 24 y old. Sodium intake was higher among males, adults between 25 and 44 y old, and adults with a higher BMI. The mean sodium-to-potassium ratio was 1.7 mg/mg overall and highest among Black females between 18 and 64 y old and Black and Latino males between 18 and 44 y old.

Our observed lack of change in sodium intake between 2010 and 2018 is consistent with other United States-based studies that have shown no change in the sodium intake of adults over the last 20 y [28,29]. Significant sources of sodium include commercially processed and packaged foods [30] and foods prepared in fast-food and full-service restaurants [31,32]. We did not observe an association between sodium intake and the frequency of eating meals prepared outside the home, which is consistent with people consuming high-sodium foods prepared both in and outside the home. There were several initiatives that aimed to reduce population-level sodium. For example, the NSRI set voluntary sodium reduction targets for packaged and restaurant foods, and the sodium warning rule [15] mandated that chain restaurants display icons that warn customers of high-sodium menu items and associated health risks. We did not observe reductions in sodium intake across any category of eating-out frequency, including those who eat exclusively at home or those who mostly eat out. Although a modest decline in sodium content across packaged food categories was observed soon after the launch of NSRI [33], it was lower than the goal that was set (7% compared with 25%) and a recent evaluation showed that industry reduction of sodium did not continue to decrease beyond 2014 [34]. A separate evaluation showed that the number of high-sodium menu items did not decrease 1 y after implementation of the sodium icon warning [35]. We also found that a third of participants reported that they looked for sodium information at least most of the time suggesting awareness regarding sodium in some of the NYC adult population. However, the frequency of using it most of the time decreased between 2010 and 2018, which suggests that in 2018, there was decreased population-level knowledge about sodium as others have found a positive association between the use of Nutrition Facts labels and knowing about sodium [36].

We found that sodium intake changed from 2010 to 2018 by age. The most prominent change was the decrease in sodium intake by 500–700 mg/d among adults 18–24 y. Temporal declines in sodium intake have been observed nationally among adults aged 19–30 y from 2003 to 2016, but the findings were no longer significant once total energy intake was adjusted for [29]. A separate study showed no difference in sodium intake between the 2005–2006 and 2015–2016 cycles of the NHANES among adults 18–29 y old with and without energy adjustment [28]. Our study methods did not allow for the measurement of total caloric intake in either round of HFUS. The observed decreases may also reflect demographic, social, or cultural changes in NYC’s population over the last decade, although we controlled for some of these factors in regression analysis. Lastly, it is unclear whether the sodium-reduction initiatives played a role as they were meant to be citywide and not targeted at any 1 group.

Although we found that the average intake exceeded recommendations among all groups examined, sodium intake was higher among males and adults with obesity, consistent with national data [9]. Contrary to higher sodium intake among Black and Latino/a adults reported from the 2010 HFUS [12], there were no differences by race and ethnicity in 2018. However, our assessment of the sodium-to-potassium ratio indicated much higher sodium intake and lower potassium intake among Black females between the ages of 18–64 and Black males, as well as Latino males, between the ages of 18–44. Although we did not compare sodium-to-potassium ratios between 2018 and 2010 in the current study, we previously reported that the ratios were higher among Black and Latino New Yorkers in 2010 [37]. Socioeconomic and geographic factors, many of which have been driven by discriminatory practices that result in residential segregation and barriers to resources, have limited access to healthy foods, particularly for predominantly Black [19,38,39] and Latino/a [40] communities. Given that the joint effects of high sodium and low potassium intake may more strongly predict blood pressure [41] and CVD risk [42] than either nutrient alone, the disparate sodium to potassium ratios, if left unaddressed, will continue to hinder efforts to reduce racial and ethnic inequities in health.

Our study had several limitations. We could not account for total caloric intake that could have masked or overstated changes in sodium intake. Furthermore, a single 24-h urine measurement may not reflect a person’s typical daily intake of sodium. Additionally, the planned sample size was underpowered for stratified analysis by race and ethnicity, sex, and age to understand whether factors such as frequency of eating out, use of the Nutrition Facts panel, or socioeconomic factors differed across these groups and over time. We were also not able to disaggregate the racial and ethnic groups which each include diverse ethnicities, cultures, and histories that are important for shaping dietary patterns and behaviors. Although many of our findings are consistent with national data, results from our NYC-based study may not be generalizable to other cities and towns across the United States because of different underlying populations, food environments, policies, and sodium-specific initiatives. The survey also did not include contextual questions around broader knowledge of sodium, motivation to reduce sodium, and barriers to choosing lower sodium foods in both the retail and restaurant setting. Lastly, changes to the hypertension question may have introduced some misclassification bias to how we captured those with a history of CVD each year. Despite these limitations, our population-based study used 24-h urine collection to better assess sodium intake among NYC adults at 2 timepoints almost a decade apart and provides an additional timepoint to assess future measures against.

We found that sodium intake has not decreased overall and is still higher than the sodium CDRR of 2300 mg/d. Additionally, there are groups, notably Black females and younger Black and Latino males, whose sodium intake is high and potassium intake is low. Efforts aimed at individual-level modification of sodium intake are not enough as sodium is present in much of the food supply and access to foods high in sodium or potassium is inequitably distributed. At the same time, current salt reduction initiatives have achieved little progress in reducing sodium intake [43]. Although sodium reduction strategies can be successful, there are a few factors to consider. An intervention in China, where food consumed at home is the primary source of sodium intake, may have been successful because it is easier to reach individuals rather than large industries [44]. The United Kingdom, where the main sources of sodium more closely match that of the United States, demonstrated that comprehensive sodium reduction interventions targeted at industry can reduce the intake of sodium, but the improvements can also be reversed if the programs and policies are not sustained [45]. In 2021, the FDA took steps toward sodium reduction in the food supply by introducing near-term voluntary sodium reduction guidance [46], but more action is urgently needed—CVD continues to be a leading cause of death and contributes to significant racial and ethnic disparities. Subsequent additional sodium reduction guidance is warranted to encourage an industry-wide reduction of sodium in packaged and restaurant foods; mandatory guidance should also be considered. This should be paired with continuous monitoring and public reporting of sodium in the food supply in addition to exploring new and innovative policies to create a healthier food environment. Policies and programs that increase access to and affordability of healthier, potassium-rich foods, like fruits and vegetables, are also needed to improve the overall diet quality of all New Yorkers.

## Acknowledgments

We thank the following colleagues for their thoughtful contributions: Sungwoo Lim for statistical guidance and Kim Kessler and Shadi Chamany for comments on the manuscript.

## Author contributions

The authors’ responsibilities were as follows – CD, JPJ: designed research; ALS: conducted research; CD, DP: analyzed data; CD, AS, ES, JPJ: wrote the article; CD: had primary responsibility for the final content; and all authors: read and approved the final manuscript.

## Conflict of interest

The authors declare that they have no known competing financial interests or personal relationships that could have appeared to influence the work reported in this article.

## Funding

The 2010 HFUS was made possible by funding from the 10.13039/100000867Robert Wood Johnson Foundation (grant Pioneer #65934), the 10.13039/100019230New York State Health Foundation (grant #2009-3021329), the 10.13039/100021816National Association of County & City Health Officials, the 10.13039/100000030Centers for Disease Control and Prevention (grant 5U38HM000449-02), the 10.13039/100007746W.K. Kellogg Foundation, and the 10.13039/100000016United States Department of Health and Human Services. This funding was administered by the Fund for Public Health in New York, a private nonprofit organization that supports innovative initiatives of the 10.13039/100004851New York City Department of Health and Mental Hygiene. The New York City Department of Health also partially funded the 2010 HFUS and fully funded the 2018 HFUS. The supporting sources were not involved in the study design, collection, analysis, interpretation of data, or writing of the report and had no restrictions regarding the submission of the report for publication.

## Data availability

Data described in the manuscript, code book, and analytic code will be made available upon request pending application and approval.

## References

[1] Nestel P.J., Mori T.A. (2022). Dietary patterns, dietary nutrients and cardiovascular disease. Rev. Cardiovasc. Med..

[2] Li W., Onyebeke C., Huynh M., Castro A., Falci L., Gurung S. (2019).

[3] Virani S.S., Alonso A., Benjamin E.J., Bittencourt M.S., Callaway C.W., Carson A.P. (2020). Heart disease and stroke statistics-2020 update: a report from the American Heart Association. Circulation.

[4] Fuchs F.D., Whelton P.K. (2020). High blood pressure and cardiovascular disease. Hypertension.

[5] Campbell N.R.C., Whelton P.K., Orias M., Wainford R.D., Cappuccio F.P., Ide N. (2023). 2022 World Hypertension League, resolve to save lives and International Society of Hypertension dietary sodium (salt) global call to action. J. Hum. Hypertens..

[6] Houston M.C. (2011). The importance of potassium in managing hypertension. Curr. Hypertens. Rep..

[7] U.S. Department of Health and Human Services and U.S. Department of Agriculture. 2020–2025 Dietary Guidelines for Americans [Internet]. 9th Edition [Updated December 2020] Available from: https://www.dietaryguidelines.gov/sites/default/files/2021-03/Dietary_Guidelines_for_Americans-2020-2025.pdf.

[8] National Academies of Sciences, Engineering, and Medicine (2019).

[9] Cogswell M.E., Loria C.M., Terry A.L., Zhao L., Wang C.Y., Chen T.C. (2018). Estimated 24-hour urinary sodium and potassium excretion in US adults. JAMA.

[10] Bentley B. (2006). A review of methods to measure dietary sodium intake. J. Cardiovasc. Nurs..

[11] Rhodes D.G., Murayi T., Clemens J.C., Baer D.J., Sebastian R.S., Moshfegh A.J. (2013). The USDA automated multiple-pass method accurately assesses population sodium intakes. Am. J. Clin. Nutr..

[12] Angell S.Y., Yi S., Eisenhower D., Kerker B.D., Curtis C.J., Bartley K. (2014). Sodium intake in a cross-sectional, representative sample of New York City adults. Am. J. Public Health..

[13] US Food and Drug Administration (2011). Approaches to reducing sodium consumption; establishment of dockets; request for comments, data, and information; extension of comment period. Fed Regist.

[14] Voluntary Sodium Reduction Goals (2016). Target Mean and Upper Bound Concentrations for Sodium in Commercially Processed, Packaged, and Prepared Foods; Draft Guidance for Industry; Availability. Fed Regist.

[15] Anekwe A.V., Lent M., Farley S.M., Kessler K., Kennelly M.O., Angell S.Y. (2019). New York city’s sodium warning regulation: from conception to enforcement. Am. J. Public Health..

[16] Loftfield E., Yi S., Curtis C.J., Bartley K., Kansagra S.M. (2013). Potassium and fruit and vegetable intakes in relation to social determinants and access to produce in New York City. Am. J. Clin. Nutr..

[17] Churchwell K., Elkind M.S.V., Benjamin R.M., Carson A.P., Chang E.K., Lawrence W. (2020). Call to action: structural racism as a fundamental driver of health disparities: a presidential advisory from the American Heart Association. Circulation.

[18] Zenk S.N., Powell L.M., Rimkus L., Isgor Z., Barker D.C., Ohri-Vachaspati P. (2014). Relative and absolute availability of healthier food and beverage alternatives across communities in the United States. Am. J. Public Health..

[19] Landrine H., Corral I. (2009). Separate and unequal: residential segregation and black health disparities. Ethn. Dis..

[20] Larson N.I., Story M.T., Nelson M.C. (2009). Neighborhood environments: disparities in access to healthy foods in the U.S. Am. J. Prev. Med..

[21] Ogunniyi M.O., Commodore-Mensah Y., Ferdinand K.C. (2021). Race, ethnicity, hypertension, and heart disease: JACC focus seminar 1/9. J. Am. Coll. Cardiol..

[22] Dominianni C., Seltzer B. (January 2023). Hypertension prevalence, awareness, treatment, and control in New York City [Internet]. New York City Department of Health and Mental Hygiene: Epi Data Brief.

[23] Rana J., Oldroyd J., Islam M.M., Tarazona-Meza C.E., Islam R.M. (2020). Prevalence of hypertension and controlled hypertension among United States adults: Evidence from NHANES 2017-18 survey. Int. J. Cardiol. Hypertens.

[24] Mirmiran P., Gaeini Z., Bahadoran Z., Ghasemi A., Norouzirad R., Tohidi M. (2021). Urinary sodium-to-potassium ratio: a simple and useful indicator of diet quality in population-based studies. Eur. J. Med. Res..

[25] New York City Department of Health and Mental Hygiene. Community Health Survey methodology - NYC health [Internet] [July 12, 2023]. Available from: https://www.nyc.gov/site/doh/data/data-sets/community-health-survey-methodology.page.

[26] National Health and Nutrition Examination Survey. Physician examination procedures manual [Internet] [August 11, 2022]. Available from: https://wwwn.cdc.gov/nchs/data/nhanes/2007-2008/manuals/manual_pe.pdf.

[27] Sanderson M Y.S., Bartley K., Quitoni K. (2012). The Community Health Survey, Heart Follow-Up Study Methodology Report. The New York City Department of Health and Mental Hygiene.

[28] Hu J.R., Sahni S., Mukamal K.J., Millar C.L., Wu Y., Appel L.J. (2020). Dietary sodium intake and sodium density in the United States: estimates from NHANES 2005-2006 and 2015-2016. Am. J. Hypertens..

[29] Clarke L.S., Overwyk K., Bates M., Park S., Gillespie C., Cogswell M.E. (2021). Temporal trends in dietary sodium intake among adults aged ≥19 Years – United States, 2003–2016. MMWR. Morb. Mortal. Wkly. Rep..

[30] Harnack L.J., Cogswell M.E., Shikany J.M., Gardner C.D., Gillespie C., Loria C.M. (2017). Sources of sodium in US adults from 3 geographic regions. Circulation.

[31] An R. (2016). Fast-food and full-service restaurant consumption and daily energy and nutrient intakes in US adults. Eur. J. Clin. Nutr..

[32] Wellard-Cole L., Davies A., Allman-Farinelli M. (2022). Contribution of foods prepared away from home to intakes of energy and nutrients of public health concern in adults: a systematic review. Crit. Rev. Food Sci. Nutr..

[33] Curtis C.J., Clapp J., Niederman S.A., Ng S.W., Angell S.Y. (2016). US food industry progress during the national salt reduction initiative: 2009-2014. Am. J. Public Health..

[34] Moran A.J., Wang J., Sharkey A.L., Dowling E.A., Curtis C.J., Kessler K.A. (2022). US food industry progress toward salt reduction, 2009-2018. Am. J. Public Health..

[35] Sisti J.S., Prasad D., Niederman S., Mezzacca T.A., Anekwe A.V., Clapp J. (2023). Sodium content of menu items in New York City chain restaurants following enforcement of the sodium warning icon rule, 2015-2017. PLOS ONE.

[36] Dewey G., Wickramasekaran R.N., Kuo T., Robles B. (2017). Does sodium knowledge affect dietary choices and health behaviors? Results from a survey of Los Angeles county residents. Prev. Chronic. Dis..

[37] Yi S.S., Curtis C.J., Angell S.Y., Anderson C.A., Jung M., Kansagra S.M. (2014). Highlighting the ratio of sodium to potassium in population-level dietary assessments: cross-sectional data from New York City, USA. Public Health Nutr.

[38] Gordon C., Purciel-Hill M., Ghai N.R., Kaufman L., Graham R., Van Wye G. (2011). Measuring food deserts in New York City’s low-income neighborhoods. Health Place.

[39] Bertoni A.G., Foy C.G., Hunter J.C., Quandt S.A., Vitolins M.Z., Whitt-Glover MC. (2011). A multilevel assessment of barriers to adoption of Dietary Approaches to Stop Hypertension (DASH) among African Americans of low socioeconomic status. J. Health Care Poor Underserved.

[40] Abraido-Lanza A.F., Echeverria S.E., Florez K.R. (2016). Latino immigrants, acculturation, and health: promising new directions in research. Annu. Rev. Public Health.

[41] Ndanuko R.N., Ibrahim R., Hapsari R.A., Neale E.P., Raubenheimer D., Charlton K.E. (2021). Association between the urinary sodium to potassium ratio and blood pressure in adults: a systematic review and meta-analysis. Adv. Nutr..

[42] Cook N.R., Obarzanek E., Cutler J.A., Buring J.E., Rexrode K.M., Kumanyika S.K. (2009). Joint effects of sodium and potassium intake on subsequent cardiovascular disease: the Trials of Hypertension Prevention follow-up study. Arch. Intern. Med..

[43] Santos J.A., Tekle D., Rosewarne E., Flexner N., Cobb L., Al-Jawaldeh A. (2021). A systematic review of salt reduction initiatives around the world: a midterm evaluation of progress towards the 2025 global non-communicable diseases salt reduction target. Adv. Nutr..

[44] Xu A., Ma J., Guo X., Wang L., Wu J., Zhang J. (2020). Association of a province-wide intervention with salt intake and hypertension in Shandong Province, China, 2011-2016. JAMA Intern. Med..

[45] Song J., Tan M., Wang C., Brown M.K., Pombo-Rodrigues S., MacGregor G.A. (2023). Salt intake, blood pressure and cardiovascular disease mortality in England, 2003-2018. J. Hypertens..

[46] US Food and Drug Administration. Guidance for industry: voluntary sodium reduction goals [Internet]. [updated October 2021]. Available from: https://www.fda.gov/regulatory-information/search-fda-guidance-documents/guidance-industry-voluntary-sodium-reduction-goals.

