# Supplementary material for: Sodium Intake and Sodium to Potassium Ratio among New York City Adults in the 2018 Heart Follow-Up Study
Source: Curr Dev Nutr. 2024 Mar 21;8(4):102143. doi: 10.1016/j.cdnut.2024.102143 (PMC11024494; doi:10.1016/j.cdnut.2024.102143)

**Supplementary Table 1.** Fully-adjusted weighted mean sodium intake of NYC adults (≥18 years old) from regression models using log-transformed sodium values, 2010 and 2018

|  | 2010  Weighted Mean Sodium^1^ (mg/day) | 2018  Weighted Mean Sodium^1^ (mg/day) | p-value^2^ |
| --- | --- | --- | --- |
| Total | 2901 | 2906 | 0.89 |
| Age (yrs) |  |  |  |
| 18-24 | 3579 | 2907 | 0.003 |
| 25-44 | 2924 | 3037 |  |
| 45-64 | 2930 | 2868 |  |
| ≥65 | 2413 | 2591 |  |
| Sex |  |  |  |
| Male | 3113 | 3208 | 0.16 |
| Female | 2737 | 2651 |  |
| Race/ethnicity |  |  |  |
| Asian/Pacific Islander | 2983 | 3007 | 0.44 |
| Black | 3223 | 2994 |  |
| Latino/a | 3116 | 3080 |  |
| Other | 2966 | 2963 |  |
| White | 2577 | 2686 |  |
| Country of birth |  |  |  |
| United States | 2961 | 2928 | 0.65 |
| Outside of United States | 2831 | 2854 |  |
| Household income, (% of FPL) |  |  |  |
| <200 | 2984 | 2961 | 0.56 |
| 200-399 | 2989 | 2865 |  |
| ≥400 | 2747 | 2815 |  |
| BMI (kg/m^2^) |  |  |  |
| <18.5 (underweight) | 3075 | 2551 | 0.48 |
| 18.5-24.9 (normal weight) | 2681 | 2582 |  |
| 25-29.9 (overweight) | 2823 | 2909 |  |
| ≥30 (obesity) | 3254 | 3274 |  |
| Smoking status |  |  |  |
| Never | 2870 | 2860 | 0.89 |
| Current | 3051 | 2990 |  |
| Former | 2909 | 2939 |  |
| Physically active in past 30d |  |  |  |
| Yes | 2852 | 2910 | 0.07 |
| No | 3068 | 2862 |  |
| Heavy drinking^3^ |  |  |  |
| Yes | 3148 | 2803 | 0.13 |
| No | 2892 | 2901 |  |
| Look for information about sodium when using the Nutrition Facts panel |  |  |  |
| Most of the time to always | 2852 | 2809 | 0.97 |
| Rarely to sometimes | 2869 | 2872 |  |
| Never | 2969 | 3008 |  |
| Never use the Nutrition Facts Panel | 3027 | 3042 |  |
| Eat meals purchased at deli, street vendor or restaurant |  |  |  |
| 0 meals per week | 2703 | 2837 | 0.07 |
| >0-3 meals per week | 2989 | 2848 |  |
| >3-6 meals per week | 2974 | 2981 |  |
| >6 meals per week | 2713 | 3062 |  |
| History of cardiovascular disease^4^ |  |  |  |
| Yes | 2855 | 2957 | 0.23 |
| No | 2930 | 2872 |  |

Abbreviation: FPL, federal poverty level.

^1^Exponentiated weighted means are shown. Regression models included age, sex, race/ethnicity, country of birth, household income, BMI, smoking status, physically active, heavy drinking, look for sodium when using the nutrition facts panel, eat meals purchased at deli, street vendor, or restaurant, cardiovascular disease history, and daily log-transformed potassium intake.

^2^P-values were obtained from t-test comparisons of 2010 and 2018 overall and from tests for interaction between year and all other variables.

^3^Defined as men having >2 drinks per day or women having >1 drink per day.

^4^Includes self-reported hypertension, self-reported stroke, or self-reported heart disease. Note that the 2010 definition was based on separate responses about self-reported congestive heart failure, coronary heart disease, angina pectoris, or myocardial infarction which we considered to be captured by the broader 2018 question on heart disease.

**Supplementary Table 2.** Weighted mean sodium and mean sodium to potassium ratio by sex, race and ethnicity, and age, 2018

|  |  | Sodium (mg/day) | | |  | Sodium to Potassium Ratio (mg/mg) | | |
| --- | --- | --- | --- | --- | --- | --- | --- | --- |
|  | N | Mean (SE) | p-value (set 1) | p-value (set 2) |  | Mean (SE) | p-value (set 1) | p-value (set 2) |
| Male |  |  |  |  |  |  |  |  |
| Asian, 18-44 | 53 | 3336 (306) | 0.02 | 0.27 |  | 1.7 (0.15) | 0.01 | 0.02 |
| Black, 18-44 | 108 | 3797 (282) | 0.26 | Ref |  | 2.1 (0.12) | 0.74 | Ref |
| Latino, 18-44 | 188 | 4163 (165) | Ref | 0.26 |  | 2.1 (0.07) | Ref | 0.74 |
| White, 18-44 | 116 | 3866 (264) | 0.34 | 0.86 |  | 1.6 (0.09) | <0.001 | <0.001 |
|  |  |  |  |  |  |  |  |  |
| Asian, 45-64 | 33 | 3852 (304)^1^ | 0.21 | 0.92 |  | 1.8 (0.11)^1^ | 0.46 | 0.93 |
| Black, 45-64 | 106 | 3815 (188) | 0.07 | Ref |  | 1.9 (0.08) | 0.33 | Ref |
| Latino, 45-64 | 144 | 4307 (199) | Ref | 0.07 |  | 1.7 (0.08) | Ref | 0.33 |
| White, 45-64 | 140 | 3832 (214) | 0.10 | 0.95 |  | 1.4 (0.11) | 0.02 | 0.001 |
|  |  |  |  |  |  |  |  |  |
| Asian, 65+ | 14 | 3776 (762)^1^ | 0.88 | 0.19 |  | 1.9 (0.11)^1^ | 0.03 | 0.35 |
| Black, 65+ | 36 | 2727 (231) | 0.01 | Ref |  | 1.7 (0.13)^1^ | 0.25 | Ref |
| Latino, 65+ | 58 | 3655 (249) | Ref | 0.01 |  | 1.5 (0.12) | Ref | 0.25 |
| White, 65+ | 119 | 3196 (154) | 0.12 | 0.09 |  | 1.4 (0.16) | 0.45 | 0.09 |
| Female |  |  |  |  |  |  |  |  |
| Asian, 18-44 | 54 | 2934 (166) | 0.64 | 0.55 |  | 1.8 (0.12) | 1.00 | <0.001 |
| Black, 18-44 | 112 | 3091 (202) | 0.77 | Ref |  | 2.0 (0.14) | 0.26 | Ref |
| Latino, 18-44 | 242 | 3026 (108) | Ref | 0.77 |  | 1.8 (0.07) | Ref | 0.26 |
| White, 18-44 | 105 | 2849 (156) | 0.35 | 0.34 |  | 1.4 (0.08) | <0.001 | <0.001 |
|  |  |  |  |  |  |  |  |  |
| Asian, 45-64 | 34 | 3058 (285)^1^ | 0.65 | 0.92 |  | 1.5 (0.14)^1^ | 0.70 | 0.04 |
| Black, 45-64 | 173 | 3090 (167) | 0.42 | Ref |  | 2.2 (0.29) | 0.05 | Ref |
| Latino, 45-64 | 199 | 2911 (143) | Ref | 0.42 |  | 1.6 (0.07) | Ref | 0.05 |
| White, 45-64 | 131 | 2626 (132) | 0.14 | 0.03 |  | 1.2 (0.06) | <0.001 | <0.001 |
|  |  |  |  |  |  |  |  |  |
| Asian, 65+ | 18 | 2394 (310)^1^ | 0.53 | 0.89 |  | 1.1 (0.13)^1^ | 0.14 | 0.12 |
| Black, 65+ | 57 | 2342 (212) | 0.36 | Ref |  | 1.4 (0.11) | 0.91 | Ref |
| Latino, 65+ | 68 | 2652 (267) | Ref | 0.36 |  | 1.4 (0.10) | Ref | 0.91 |
| White, 65+ | 117 | 2497 (124) | 0.60 | 0.53 |  | 1.3 (0.09) | 0.58 | 0.51 |

Abbreviation: SE, standard error.

^1^Estimate should be interpreted with caution. Estimate's relative standard error is >30% or the sample size is too small making the estimate potentially unreliable.

**Supplemental Figure 1.** Flow chart of the 2018 NYC Heart Follow-Up Study participants.


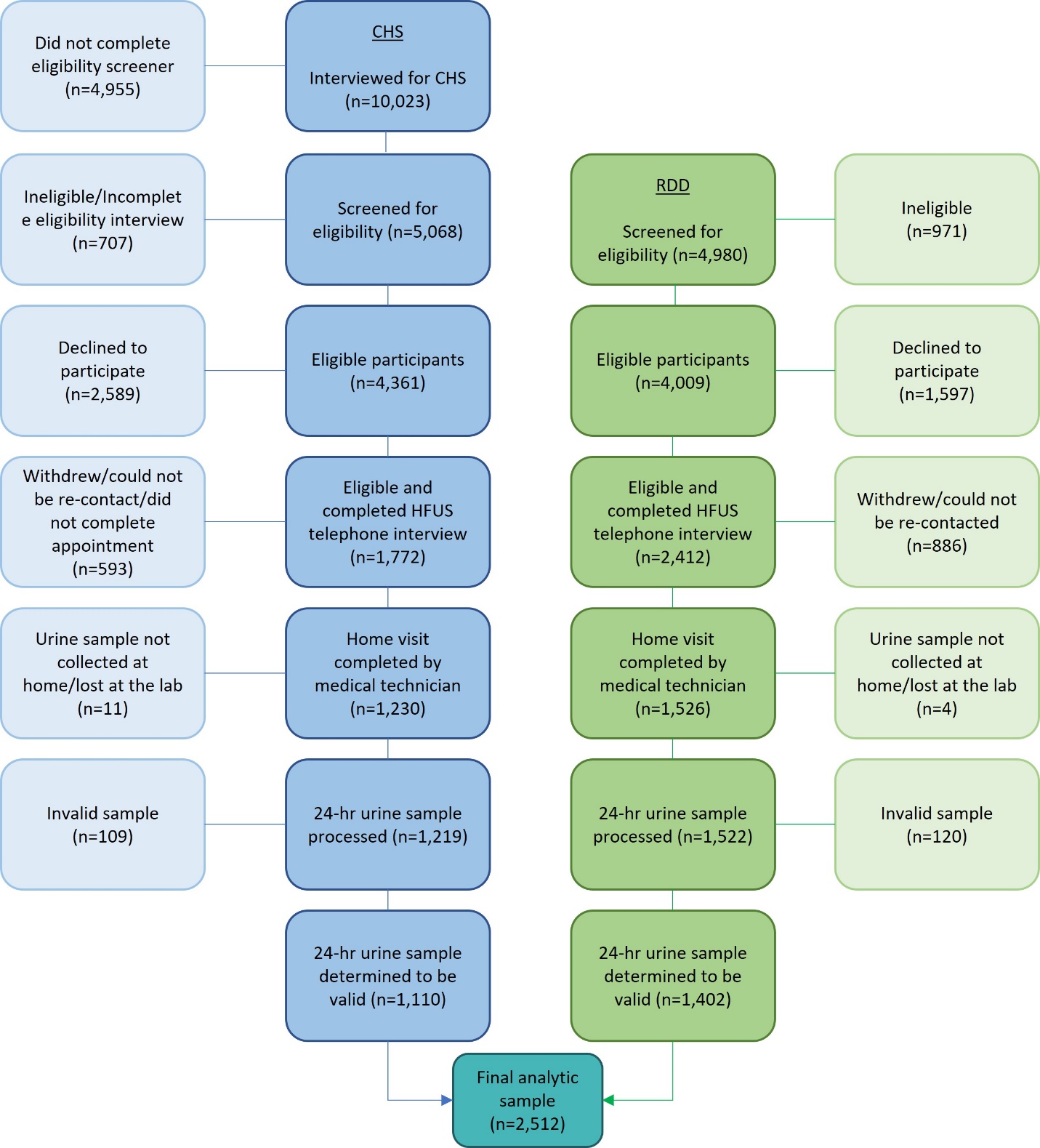

Supplement: Multimedia Component 1 [file mmc1.docx]
